# Supplementary material for: Escherichia coli Nissle 1917 Enhances Efficacy of Oral Attenuated Human Rotavirus Vaccine in a Gnotobiotic Piglet Model
Source: Vaccines (Basel). 2022 Jan 6;10(1):83. doi: 10.3390/vaccines10010083 (PMC8779073; doi:10.3390/vaccines10010083)
Supplement: Supplementary file 1 [file vaccines-10-00083-s001.zip › vaccines-1523374-supplementary.pptx]

## Slide 1
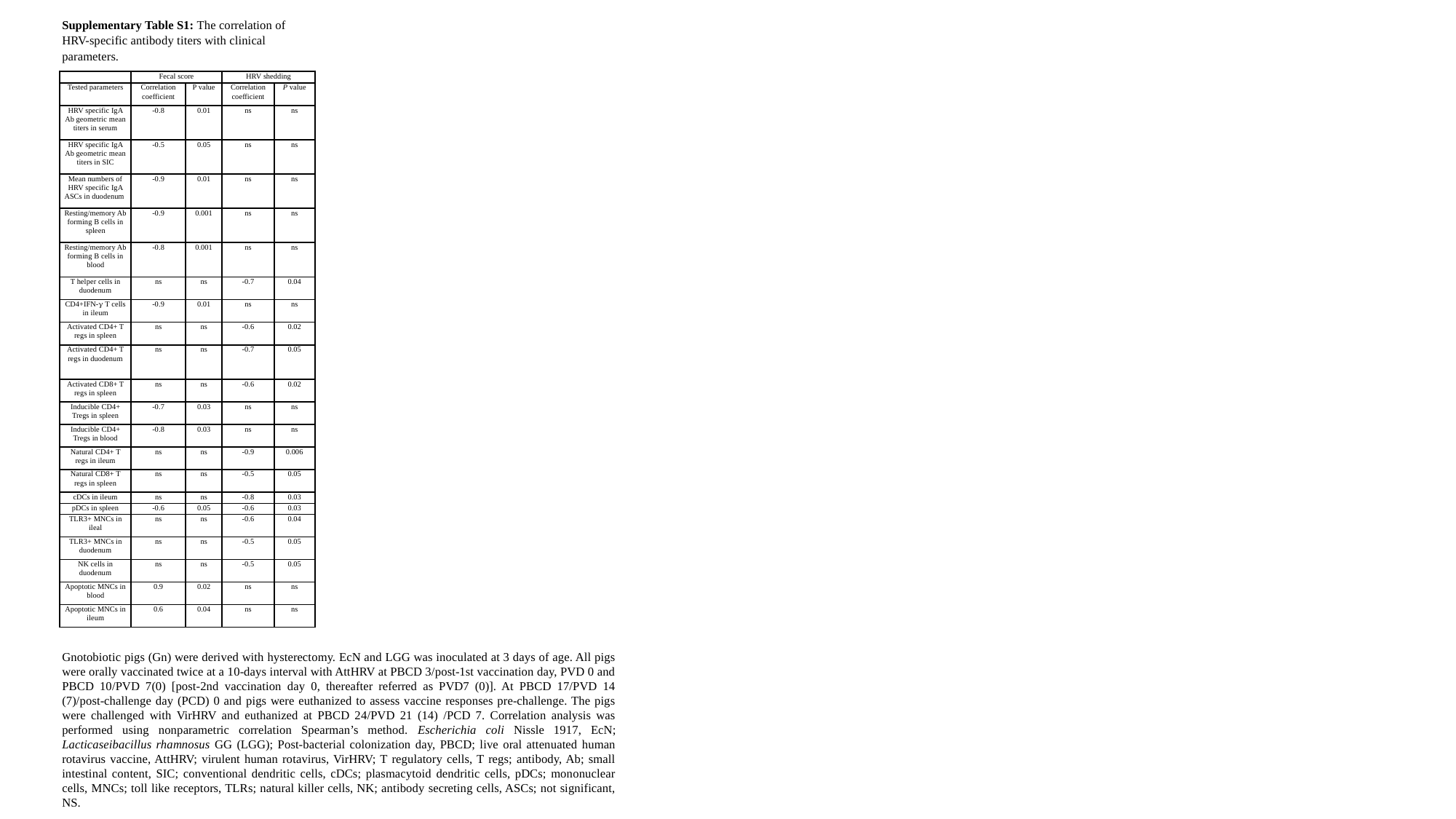

Supplementary Table S1: The correlation of HRV-specific antibody titers with clinical parameters.
| | Fecal score | | HRV shedding | |
| --- | --- | --- | --- | --- |
| Tested parameters | Correlation coefficient | P value | Correlation coefficient | P value |
| HRV specific IgA Ab geometric mean titers in serum | -0.8 | 0.01 | ns | ns |
| HRV specific IgA Ab geometric mean titers in SIC | -0.5 | 0.05 | ns | ns |
| Mean numbers of HRV specific IgA ASCs in duodenum | -0.9 | 0.01 | ns | ns |
| Resting/memory Ab forming B cells in spleen | -0.9 | 0.001 | ns | ns |
| Resting/memory Ab forming B cells in blood | -0.8 | 0.001 | ns | ns |
| T helper cells in duodenum | ns | ns | -0.7 | 0.04 |
| CD4+IFN-γ T cells in ileum | -0.9 | 0.01 | ns | ns |
| Activated CD4+ T regs in spleen | ns | ns | -0.6 | 0.02 |
| Activated CD4+ T regs in duodenum | ns | ns | -0.7 | 0.05 |
| Activated CD8+ T regs in spleen | ns | ns | -0.6 | 0.02 |
| Inducible CD4+ Tregs in spleen | -0.7 | 0.03 | ns | ns |
| Inducible CD4+ Tregs in blood | -0.8 | 0.03 | ns | ns |
| Natural CD4+ T regs in ileum | ns | ns | -0.9 | 0.006 |
| Natural CD8+ T regs in spleen | ns | ns | -0.5 | 0.05 |
| cDCs in ileum | ns | ns | -0.8 | 0.03 |
| pDCs in spleen | -0.6 | 0.05 | -0.6 | 0.03 |
| TLR3+ MNCs in ileal | ns | ns | -0.6 | 0.04 |
| TLR3+ MNCs in duodenum | ns | ns | -0.5 | 0.05 |
| NK cells in duodenum | ns | ns | -0.5 | 0.05 |
| Apoptotic MNCs in blood | 0.9 | 0.02 | ns | ns |
| Apoptotic MNCs in ileum | 0.6 | 0.04 | ns | ns |
Gnotobiotic pigs (Gn) were derived with hysterectomy. EcN and LGG was inoculated at 3 days of age. All pigs were orally vaccinated twice at a 10-days interval with AttHRV at PBCD 3/post-1st vaccination day, PVD 0 and PBCD 10/PVD 7(0) [post-2nd vaccination day 0, thereafter referred as PVD7 (0)]. At PBCD 17/PVD 14 (7)/post-challenge day (PCD) 0 and pigs were euthanized to assess vaccine responses pre-challenge. The pigs were challenged with VirHRV and euthanized at PBCD 24/PVD 21 (14) /PCD 7. Correlation analysis was performed using nonparametric correlation Spearman’s method. Escherichia coli Nissle 1917, EcN; Lacticaseibacillus rhamnosus GG (LGG); Post-bacterial colonization day, PBCD; live oral attenuated human rotavirus vaccine, AttHRV; virulent human rotavirus, VirHRV; T regulatory cells, T regs; antibody, Ab; small intestinal content, SIC; conventional dendritic cells, cDCs; plasmacytoid dendritic cells, pDCs; mononuclear cells, MNCs; toll like receptors, TLRs; natural killer cells, NK; antibody secreting cells, ASCs; not significant, NS.

## Slide 2
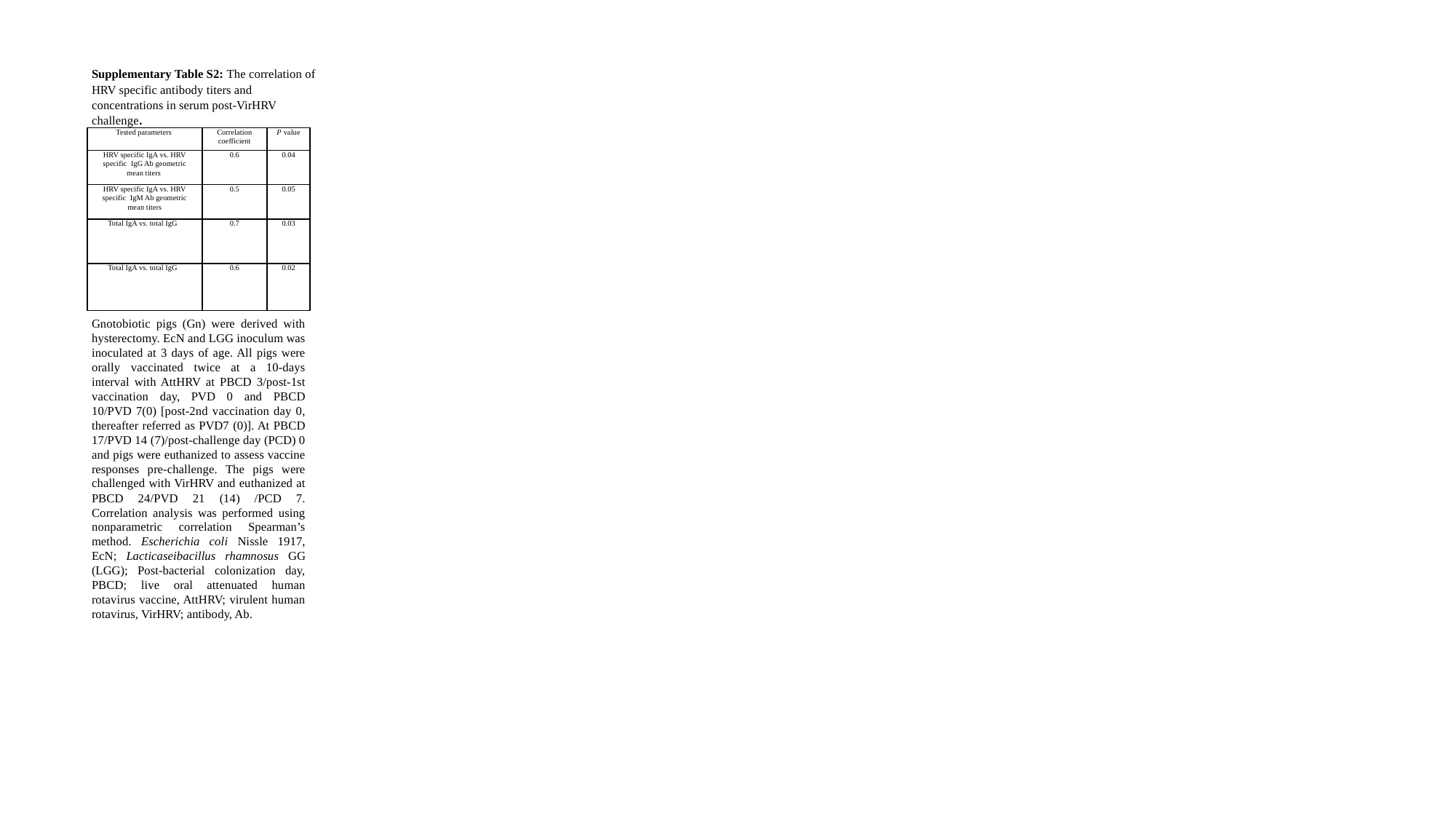

Supplementary Table S2: The correlation of HRV specific antibody titers and concentrations in serum post-VirHRV challenge.
| Tested parameters | Correlation coefficient | P value |
| --- | --- | --- |
| HRV specific IgA vs. HRV specific IgG Ab geometric mean titers | 0.6 | 0.04 |
| HRV specific IgA vs. HRV specific IgM Ab geometric mean titers | 0.5 | 0.05 |
| Total IgA vs. total IgG | 0.7 | 0.03 |
| Total IgA vs. total IgG | 0.6 | 0.02 |
Gnotobiotic pigs (Gn) were derived with hysterectomy. EcN and LGG inoculum was inoculated at 3 days of age. All pigs were orally vaccinated twice at a 10-days interval with AttHRV at PBCD 3/post-1st vaccination day, PVD 0 and PBCD 10/PVD 7(0) [post-2nd vaccination day 0, thereafter referred as PVD7 (0)]. At PBCD 17/PVD 14 (7)/post-challenge day (PCD) 0 and pigs were euthanized to assess vaccine responses pre-challenge. The pigs were challenged with VirHRV and euthanized at PBCD 24/PVD 21 (14) /PCD 7. Correlation analysis was performed using nonparametric correlation Spearman’s method. Escherichia coli Nissle 1917, EcN; Lacticaseibacillus rhamnosus GG (LGG); Post-bacterial colonization day, PBCD; live oral attenuated human rotavirus vaccine, AttHRV; virulent human rotavirus, VirHRV; antibody, Ab.

## Slide 3
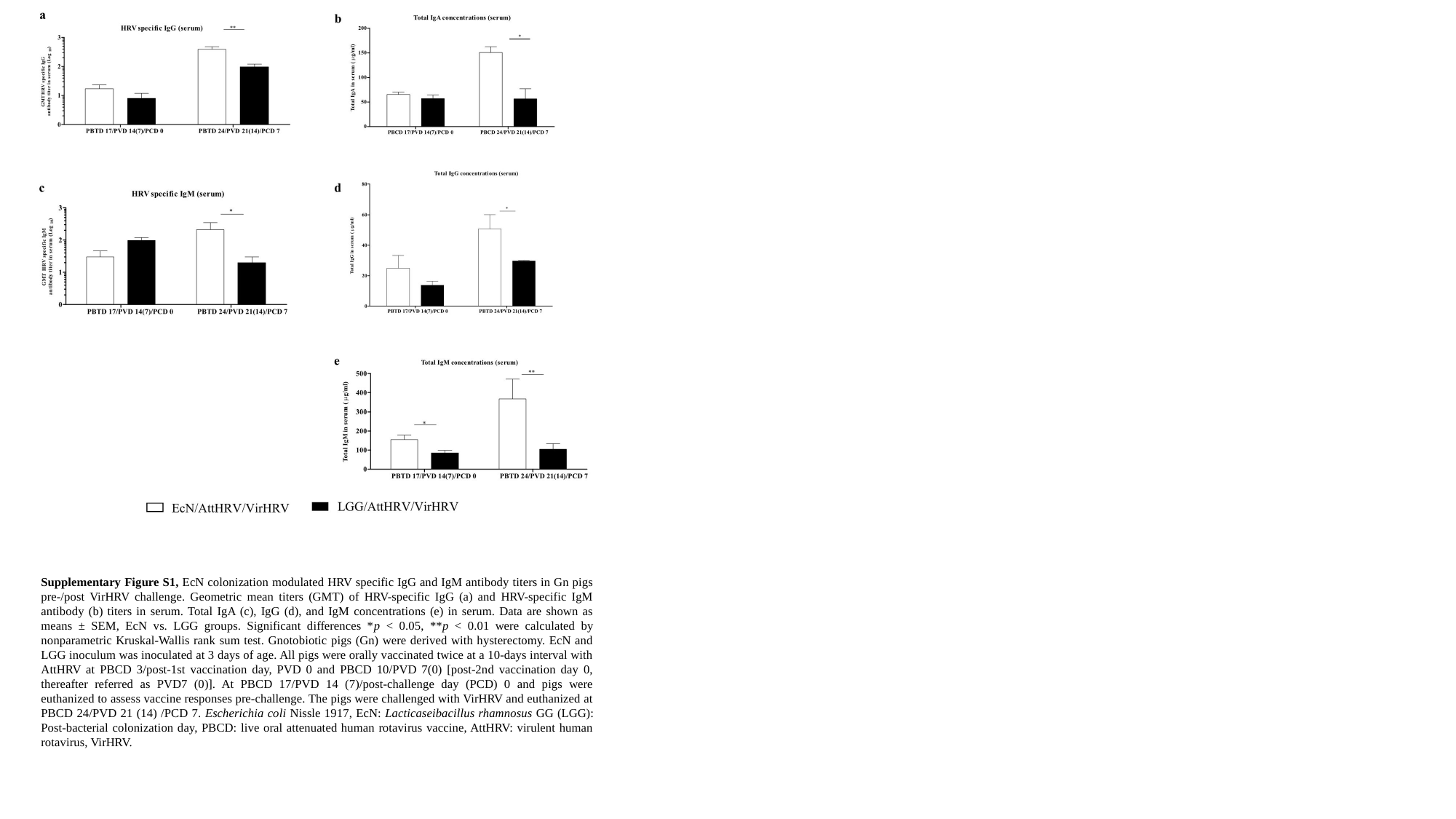

Supplementary Figure S1, EcN colonization modulated HRV specific IgG and IgM antibody titers in Gn pigs pre-/post VirHRV challenge. Geometric mean titers (GMT) of HRV-specific IgG (a) and HRV-specific IgM antibody (b) titers in serum. Total IgA (c), IgG (d), and IgM concentrations (e) in serum. Data are shown as means ± SEM, EcN vs. LGG groups. Significant differences *p < 0.05, **p < 0.01 were calculated by nonparametric Kruskal-Wallis rank sum test. Gnotobiotic pigs (Gn) were derived with hysterectomy. EcN and LGG inoculum was inoculated at 3 days of age. All pigs were orally vaccinated twice at a 10-days interval with AttHRV at PBCD 3/post-1st vaccination day, PVD 0 and PBCD 10/PVD 7(0) [post-2nd vaccination day 0, thereafter referred as PVD7 (0)]. At PBCD 17/PVD 14 (7)/post-challenge day (PCD) 0 and pigs were euthanized to assess vaccine responses pre-challenge. The pigs were challenged with VirHRV and euthanized at PBCD 24/PVD 21 (14) /PCD 7. Escherichia coli Nissle 1917, EcN: Lacticaseibacillus rhamnosus GG (LGG): Post-bacterial colonization day, PBCD: live oral attenuated human rotavirus vaccine, AttHRV: virulent human rotavirus, VirHRV.

## Slide 4
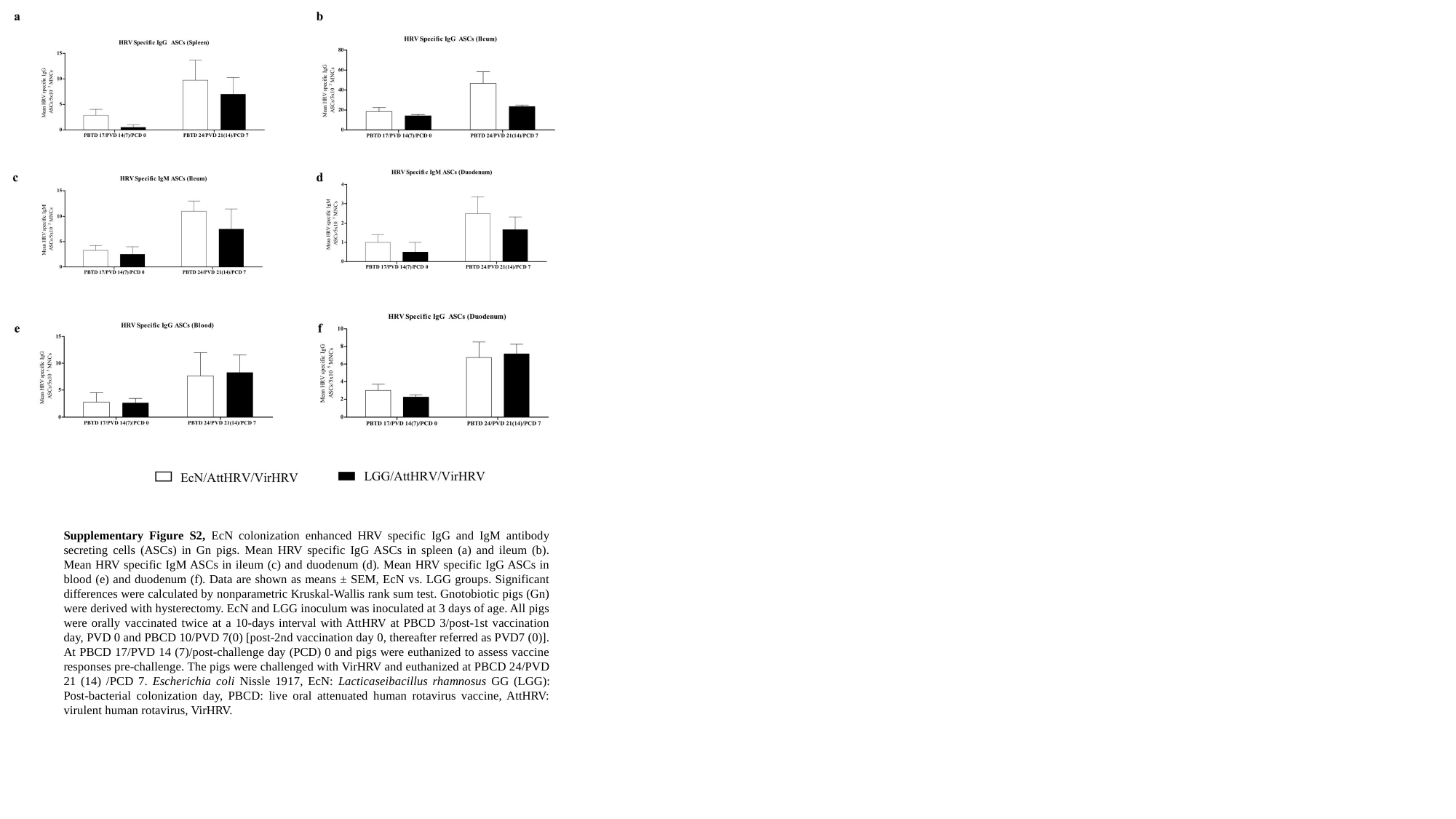

Supplementary Figure S2, EcN colonization enhanced HRV specific IgG and IgM antibody secreting cells (ASCs) in Gn pigs. Mean HRV specific IgG ASCs in spleen (a) and ileum (b). Mean HRV specific IgM ASCs in ileum (c) and duodenum (d). Mean HRV specific IgG ASCs in blood (e) and duodenum (f). Data are shown as means ± SEM, EcN vs. LGG groups. Significant differences were calculated by nonparametric Kruskal-Wallis rank sum test. Gnotobiotic pigs (Gn) were derived with hysterectomy. EcN and LGG inoculum was inoculated at 3 days of age. All pigs were orally vaccinated twice at a 10-days interval with AttHRV at PBCD 3/post-1st vaccination day, PVD 0 and PBCD 10/PVD 7(0) [post-2nd vaccination day 0, thereafter referred as PVD7 (0)]. At PBCD 17/PVD 14 (7)/post-challenge day (PCD) 0 and pigs were euthanized to assess vaccine responses pre-challenge. The pigs were challenged with VirHRV and euthanized at PBCD 24/PVD 21 (14) /PCD 7. Escherichia coli Nissle 1917, EcN: Lacticaseibacillus rhamnosus GG (LGG): Post-bacterial colonization day, PBCD: live oral attenuated human rotavirus vaccine, AttHRV: virulent human rotavirus, VirHRV.

## Slide 5
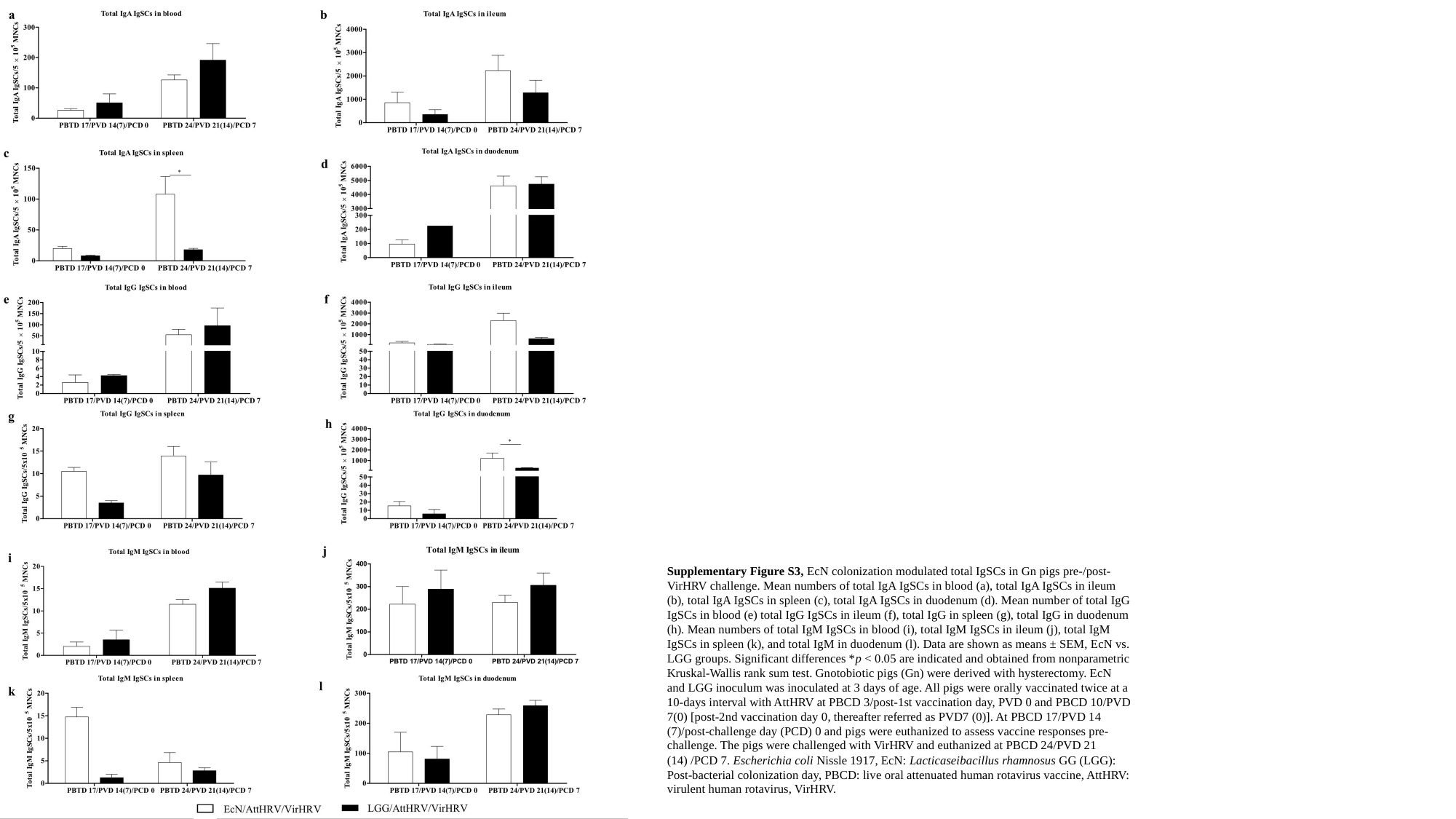

Supplementary Figure S3, EcN colonization modulated total IgSCs in Gn pigs pre-/post-VirHRV challenge. Mean numbers of total IgA IgSCs in blood (a), total IgA IgSCs in ileum (b), total IgA IgSCs in spleen (c), total IgA IgSCs in duodenum (d). Mean number of total IgG IgSCs in blood (e) total IgG IgSCs in ileum (f), total IgG in spleen (g), total IgG in duodenum (h). Mean numbers of total IgM IgSCs in blood (i), total IgM IgSCs in ileum (j), total IgM IgSCs in spleen (k), and total IgM in duodenum (l). Data are shown as means ± SEM, EcN vs. LGG groups. Significant differences *p < 0.05 are indicated and obtained from nonparametric Kruskal-Wallis rank sum test. Gnotobiotic pigs (Gn) were derived with hysterectomy. EcN and LGG inoculum was inoculated at 3 days of age. All pigs were orally vaccinated twice at a 10-days interval with AttHRV at PBCD 3/post-1st vaccination day, PVD 0 and PBCD 10/PVD 7(0) [post-2nd vaccination day 0, thereafter referred as PVD7 (0)]. At PBCD 17/PVD 14 (7)/post-challenge day (PCD) 0 and pigs were euthanized to assess vaccine responses pre-challenge. The pigs were challenged with VirHRV and euthanized at PBCD 24/PVD 21 (14) /PCD 7. Escherichia coli Nissle 1917, EcN: Lacticaseibacillus rhamnosus GG (LGG): Post-bacterial colonization day, PBCD: live oral attenuated human rotavirus vaccine, AttHRV: virulent human rotavirus, VirHRV.

## Slide 6
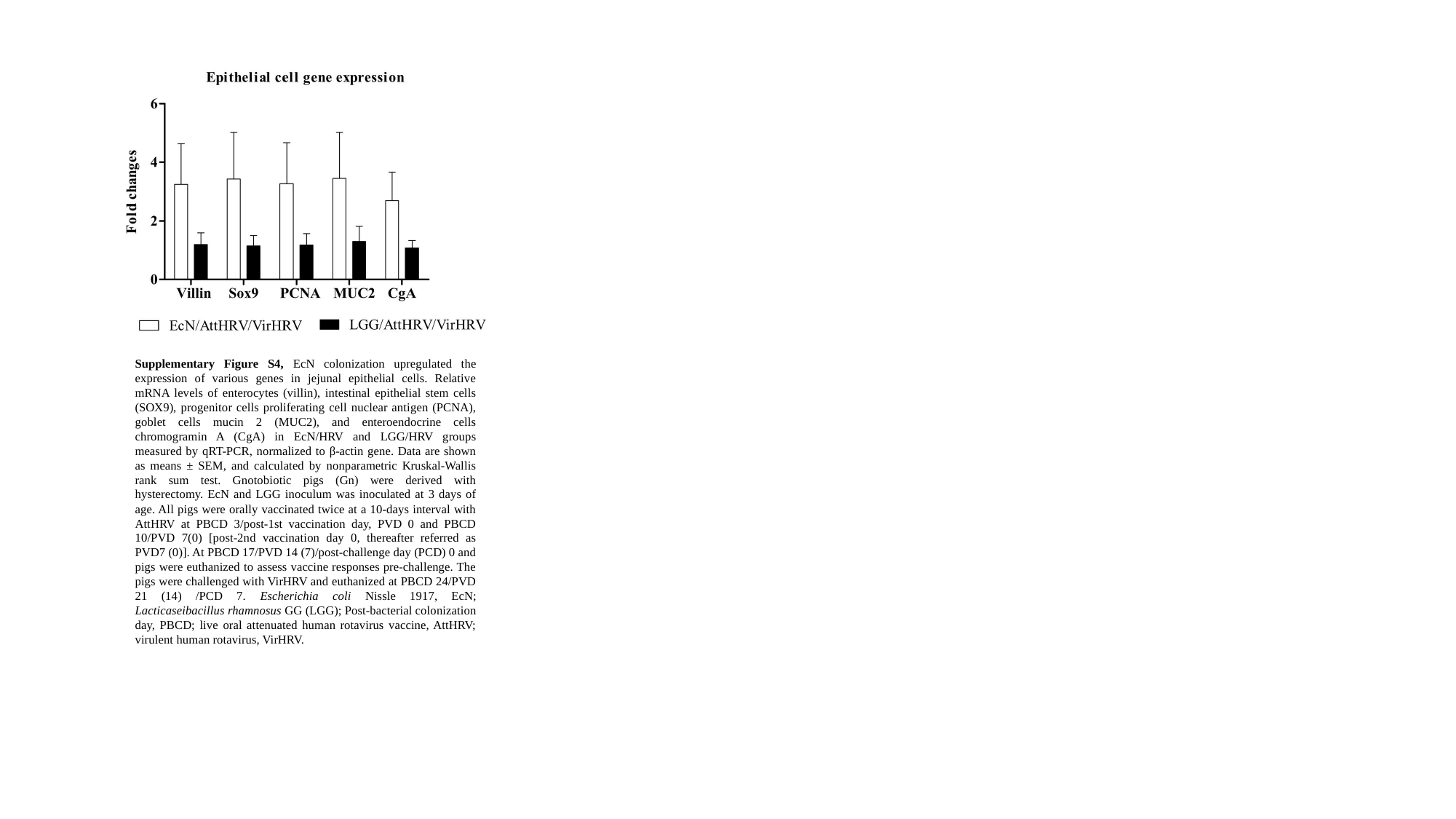

Supplementary Figure S4, EcN colonization upregulated the expression of various genes in jejunal epithelial cells. Relative mRNA levels of enterocytes (villin), intestinal epithelial stem cells (SOX9), progenitor cells proliferating cell nuclear antigen (PCNA), goblet cells mucin 2 (MUC2), and enteroendocrine cells chromogramin A (CgA) in EcN/HRV and LGG/HRV groups measured by qRT-PCR, normalized to β-actin gene. Data are shown as means ± SEM, and calculated by nonparametric Kruskal-Wallis rank sum test. Gnotobiotic pigs (Gn) were derived with hysterectomy. EcN and LGG inoculum was inoculated at 3 days of age. All pigs were orally vaccinated twice at a 10-days interval with AttHRV at PBCD 3/post-1st vaccination day, PVD 0 and PBCD 10/PVD 7(0) [post-2nd vaccination day 0, thereafter referred as PVD7 (0)]. At PBCD 17/PVD 14 (7)/post-challenge day (PCD) 0 and pigs were euthanized to assess vaccine responses pre-challenge. The pigs were challenged with VirHRV and euthanized at PBCD 24/PVD 21 (14) /PCD 7. Escherichia coli Nissle 1917, EcN; Lacticaseibacillus rhamnosus GG (LGG); Post-bacterial colonization day, PBCD; live oral attenuated human rotavirus vaccine, AttHRV; virulent human rotavirus, VirHRV.
